# Reactive Oxygen Species Cause Exercise-Induced Angina in a Myocardial Ischaemia-Reperfusion Injury Model

**DOI:** 10.3390/ijms23052820

**Published:** 2022-03-04

**Authors:** Xiaohang Wang, Hirosato Kanda, Takeshi Tsujino, Yoko Kogure, Feng Zhu, Satoshi Yamamoto, Taichi Sakaguchi, Koichi Noguchi, Yi Dai

**Affiliations:** 1 Department of Cardiovascular Surgery, Hyogo College of Medicine, Nishinomiya 663-8501, Hyogo, Japan; wangxh@huhs.ac.jp (X.W.); t-sakaguchi@hyo-med.ac.jp (T.S.); 2 Department of Pharmacy, School of Pharmacy, Hyogo University of Health Sciences, Kobe 650-8530, Hyogo, Japan; kanda@huhs.ac.jp (H.K.); ttsujino@huhs.ac.jp (T.T.); y-kogure@huhs.ac.jp (Y.K.); zhuf@huhs.ac.jp (F.Z.); syamamot@huhs.ac.jp (S.Y.); 3 Department of Anatomy and Neuroscience, Hyogo College of Medicine, Nishinomiya 663-8501, Hyogo, Japan; noguchi@hyo-med.ac.jp; 4 Department of Cardiovascular and Renal Medicine, Hyogo College of Medicine, Nishinomiya 663-8501, Hyogo, Japan

**Keywords:** angina post PCI, exercise-induced cardiac pain, p-ERK, hydrogen peroxide, TRPA1, myocardial I/R injury

## Abstract

Percutaneous coronary intervention (PCI) effectively treats obstructive coronary artery syndrome. However, 30–40% patients continue to have angina after a successful PCI, thereby reducing patient satisfaction. The mechanisms underlying persistent angina after revascularisation therapy are still poorly understood; hence, the treatment or guideline for post-PCI angina remains unestablished. Thus, this study aimed to investigate the mechanisms underlying effort angina in animals following myocardial ischaemia-reperfusion (I/R) injury. Phosphorylated extracellular signal-regulated kinase (p-ERK), a marker for painful stimulation-induced neuronal activation, was used for the investigation. After a forced treadmill exercise (FTE), the number of p-ERK-expressing neurons increased in the superficial dorsal horn of the I/R model animals. Moreover, FTE evoked hydrogen peroxide (H_2_O_2_) production in the I/R-injured heart, inducing angina through TRPA1 activation on cardiac sensory fibres. Notably, the treatment of a TEMPOL, a reactive oxygen species scavenger, or TRPA1^−/−^ mice successfully alleviated the FTE-induced p-ERK expression in the dorsal horn. The production of H_2_O_2_, a reactive oxygen species, through physical exercise contributes to angina development following I/R. Hence, our findings may be useful for understanding and treating angina following revascularisation therapy.

## 1. Introduction

Percutaneous coronary intervention (PCI) is a nonsurgical revascularisation strategy that has been effective in treating obstructive coronary artery disease (CAD), especially for ST-segment elevation myocardial infarction (STEMI). Although PCI significantly improves the clinical outcomes of patients with CAS, approximately 30–40% of these patients still suffer from persistent or recurrent angina after a successful PCI, leading to reduced patient satisfaction [1,2,3]. Recently, Crea et al. reviewed the clinical significance of angina post-PCI and suggested that the total healthcare costs of patients with persistent or recurrent angina after PCI in the first year were 1.8 times greater than those with the usual angina [4]. In addition, guidelines for the diagnosis or treatment of chest discomfort symptoms after PCI remain unestablished [1].

An important issue about persistent or recurrent angina post-PCI is whether and how cardiac ischaemia recurs in the coronary artery following PCI treatment. Risk factors responsible for cardiac ischaemia recurrence have been clinically investigated. These factors include stent thrombosis and in-stent restenosis, which have incidence rates of <1% and 5% at 1-year follow-up, respectively [5]. Moreover, they could be the cause of recurrent angina. The potential mechanism of persistent angina is coronary microvascular dysfunction based on blood flow reduction or epicardial/microvascular spasm. This putative mechanism has never been examined by basic research, and the detailed molecular mechanisms underlying persistent angina have remained unclear. Importantly, cardiac pain from stable angina is a warning sign of myocardial infarction, acting as a safety alarm in the daily lives of patients with CAD. For a better quality of life, as well as for healthcare cost reduction, medical treatment is necessary to control persistent angina occurring after a successful PCI.

Angina is induced by imbalance between myocardial oxygen supply and demand. It evokes cardiac pain or discomfort, such as squeezing, burning, and tightness. This pain sensation is elicited by spinal cardiac afferent fibres originating from dorsal root ganglions (DRGs) [6,7]. The nociceptive C-fibres of spinal cardiac afferent are distributed in the ventricle, expressing many ion channels or receptors, such as acid-sensing ion channels, transient receptor potential vanilloid-1 (TRPV1), and P2X purinergic receptors [8,9,10]. Although these molecules might contribute to the processing of general cardiac nociception, most experiments have never elucidated the mechanism of angina. The transient receptor potential ankyrin-1 (TRPA1) channel, which is a nonselective cation channel, is abundantly expressed in the nociceptive sensory neurons in DRG, serving as a pain sensor in the peripheral nervous system [11]. Particularly, TRPA1 is involved in the generation of chemically induced pain and is activated by reactive oxygen species (ROS) such as hydrogen peroxide (H_2_O_2_), hydroxyl radicals, and 4-hydroxynonenal (4-HNE) [12]. As an important function, TRPA1 mediates hypoxia-induced dysaesthesia in the somatosensory system; however, this putative mechanism in the onset of angina remains obscure.

Reperfusion is the most effective treatment for STEMI, and revascularisation therapies, including PCI, can recover the supply of oxygen and blood flow. However, blood flow restoration itself inflicts massive ischaemia-reperfusion (I/R) injury, which is common in ischaemic disease [13,14]. Although several mechanisms, such as inflammation, apoptosis, microvascular dysfunction, and oxidative stress, are involved in I/R injury [15], the relationship of angina and I/R injury is still insufficiently understood. Even though vasopressin, methacholine, and isoproterenol, which evoke vasospasm, have been used as experimental animal models for angina [16,17,18], they have less translational potential.

One of the main reasons for the delay in the elucidation of angina mechanism is the lack of established methods for evaluating cardiac pain in animals. Considering that pain is subjective, several experimental behaviour tests have been developed to detect pain. However, no behavioural test is currently available for cardiac pain evaluation. In 1999, Ji et al. reported that noxious stimulation specifically induces phosphorylated extracellular signal-regulated kinase (p-ERK) in superficial dorsal horn neurons [19]. Accumulating evidence indicates that p-ERK can be a useful marker for pain signalling in the spinal cord [20,21,22,23]. Here, we used a myocardial I/R injury animal model to study angina following revascularisation therapy for the first time. This study shows that the I/R animal model presents p-ERK expression elevation in the spinal cord after applying forced treadmill exercise (FTE). Moreover, exercise induces H_2_O_2_ production in the I/R-injured heart, thereby evoking angina through TRPA1 activation on cardiac sensory fibres. Notably, the treatment of a ROS scavenger or TRPA1^-/-^ mice successfully alleviates exercise-induced angina after an I/R injury. Hence, our study provides molecular, functional, and behavioural evidence to understand how physical activities induce angina following an I/R injury.

## 2. Results

### 2.1. Myocardial Ischaemic/Reperfusion Produces Potential Cardiac Dysfunction

**Figure 1:**
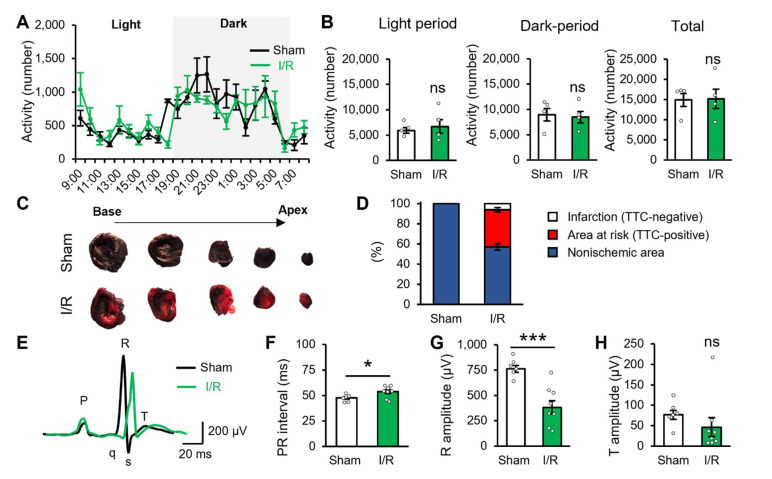
Myocardial ischaemia-reperfusion produces potential cardiac dysfunction in rats. (**A**) The average number of animal activities within 24 h in the sham group (*n* = 4, black) and I/R model group (*n* = 4, green). Shaded areas represent the dark period of the 12 h light/12 h dark cycle. (**B**) Summary of animal activities for light period (left), dark period (middle) and total activity (right). (**C**) Image shows the Evan’s blue- and 2,3,5-triphenyltetrazolium chloride (TTC)-stained heart samples of the sham group (top) and I/R model group (bottom) 2 days after the surgery. (**D**) Percentage of area at risk (red) and infarction (white) in the heart samples of the sham group (*n* = 4) and I/R model group (*n* = 4). (**E**) Sample traces illustrate the average waveform of electrocardiogram in the sham group (*n* = 6) and I/R model group (*n* = 8). (**F**–**H**) Summary of PR interval (**F**), R amplitude (**G**) and T amplitude (**H**) of the sham group (*n* = 6) and I/R model group (*n* = 8). All animals were used for the experiment 2 days after the surgery. Data are expressed as mean ± standard error of mean. * *p* < 0.05, *** *p* < 0.001, unpaired Student’s *t*-test. I/R: ischaemia-reperfusion; ns: not significant.

We used the myocardial I/R model to reproduce I/R injury in patients who underwent revascularisation therapy [15]. First, we confirmed the impact of I/R injury on daily life activities and cardiac function 2 days after the I/R surgery. Daily life activities were not affected by I/R injury in light and dark periods and in the total period of 24 h (Figure 1A,B). However, the heart of the I/R model animals showed that approximately 36.8% of its area was at risk along with a small portion (6.1%) of the infarcted area (Figure 1C,D). Moreover, cardiac function was assessed using an electrocardiogram (ECG). ECG recording showed that the PR interval was more prolonged in the I/R group compared with that of the sham group (Figure 1E,F). Additionally, the R amplitude was lower in the I/R group than in the sham group (Figure 1E,G). These ECG data suggested a decreased function in the left ventricle. Table 1 presents other ECG parameters. Taken together, these results indicated that although the I/R model animals showed normal activities of daily life, they presented cardiac dysfunctions. Meanwhile, T amplitude did not change in the I/R model group 2 days after reperfusion (Figure 1E,H), suggesting that cardiac ischaemia does not exist under the basal activity.

### 2.2. FTE Evokes Angina in I/R Model Animals

**Figure 2:**
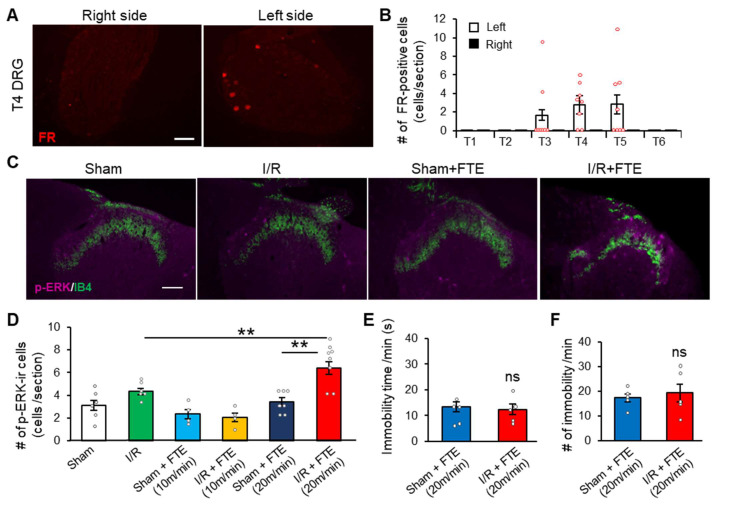
Forced treadmill exercise evokes angina in I/R model rats. (**A**) Fluorescent images show labelled DRG neurons by the intracardiac injection of 4% Fluoro-Ruby (FR) in the right side (left) and left side (right) T4 DRGs. (**B**) Distribution of FR-positive DRG neurons in the left and right DRGs among the T1–T6 levels (*n* = 8). (**C**) Double immunofluorescence histochemistry of p-ERK (magenta) and IB4 (green) in the T4–T5 dorsal horn of sham, I/R model, sham + FTE (20 m/min) and I/R model + FTE (20 m/min). Lamina II inner layer was marked by IB4. (**D**) Summary of the number of p-ERK-immunoreactive spinal neurons in lamina I–II per section in the sham group (*n* = 6), I/R model group (*n* = 6), sham + FTE group (10 m/min, *n* = 6), I/R model + FTE group (10 m/min, *n* =6), sham + FTE group (20 m/min, *n* = 6) and I/R model + FTE group (20 m/min, *n* = 8). (**E**,**F**) Time (**E**) and number (**F**) of immobility during FTE in the sham + FTE group (*n* = 6) and I/R + FTE group (*n* = 8). All animals were used for the experiments 2 days after the surgery. Scale bar = 50 μm. Data are expressed as mean ± standard error of mean. ** *p* < 0.01, one-way ANOVA with Bonferroni analysis or unpaired Student’s *t*-test. I/R, Ischaemia–reperfusion; ns, not significant; FTE, forced treadmill exercise; DRG, dorsal root ganglion; p-ERK, phosphorylated extracellular signal-regulated kinase; IB4, Isolectin B4; #, number.

In patients, a consistently high level of physical activities often triggers angina. Considering that angina is a dysaesthesia that includes chest pain or discomfort, we questioned whether physical activity could evoke angina on I/R model animals. To quantify angina in animals, we conducted an immunohistochemistry of p-ERK, which is a neuronal activation marker following noxious stimuli [20], to visualise the nociceptive processing in the dorsal horn. Firstly, we examined the distribution of cardiac sensory neurons in the thoracic DRG. The cardiac sensory neurons were labelled, and Fluoro-Ruby was injected into the left ventricular wall. We found that these cardiac sensory neurons were primarily distributed on the left side of T3–T5 DRGs (Figure 2A,B). Hence, we assessed angina by analysing p-ERK expression on the left T4–T5 dorsal horn.

Compared with sham, I/R manipulation did not affect basal p-ERK expression in the superficial dorsal horn (Figure 2C,D). Thus, the I/R model animals did not present cardiac pain with basal activity, as confirmed by the results of physical activity and T amplitude shown in Figure 1. Notably, by applying FTE (20 m/min, 10 min) on the I/R model group, we successfully detected exercise-induced cardiac pain (Figure 2C,D). The I/R + FTE group (6.4 ± 0.6 cells, *n* = 8) had more p-ERK-immunoreactive cells significantly than the sham group (3.4 ± 0.4 cells, *n* = 6) or the I/R-without-FTE group (4.3 ± 0.3 cells, *n* = 6). Exercise-induced angina was observed at least 7 days after the I/R injury (Figure S1). Furthermore, the lower intensity of FTE (10 m/min, 10 min) did not induce p-ERK expression elevation in the spinal cord (Figure 2D). The time (s) and number of immobilities during FTE were not significantly different between the sham and I/R groups (Figure 2E,F), indicating that the I/R model animals performed the same amount of exercise via FTE. Therefore, effort angina can be induced by FTE on I/R model animals, suggesting that this model is suitable and clinically relevant in the study of angina after revascularisation therapy.

### 2.3. Hydrogen Peroxide Release Provokes Exercise-Induced Angina in the I/R Model

**Figure 3:**
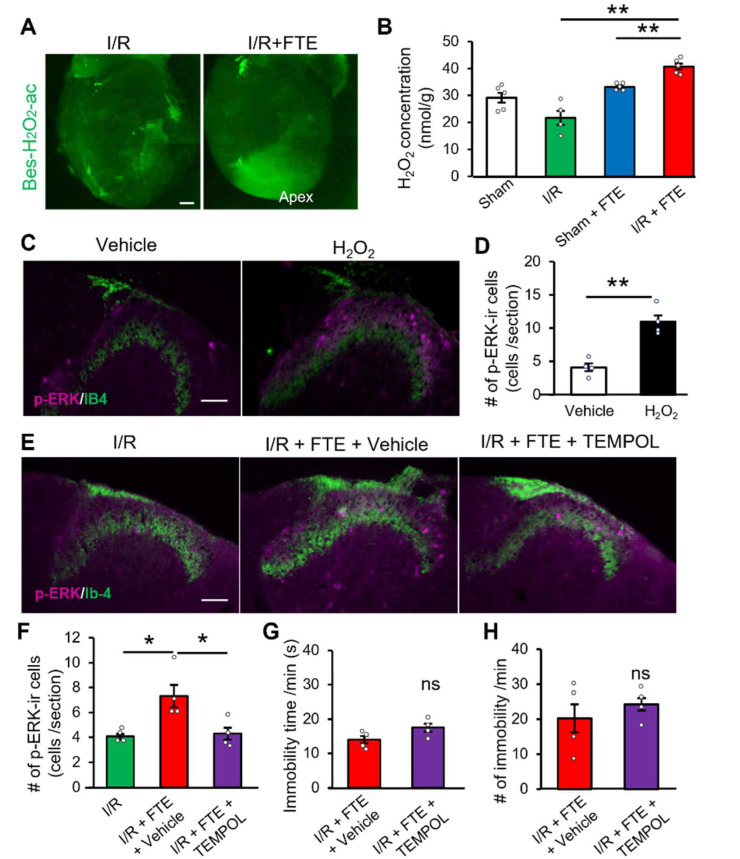
Hydrogen peroxide release mediates exercise-induced cardiac pain in I/R model rat. (**A**) Fluorescent images show the distribution of H_2_O_2_ in the whole-heart preparation of the I/R model (left) and I/R + FTE (right) detected by Bes-H_2_O_2_-ac. (**B**) Concentration of cardiac H_2_O_2_ in the sham, I/R model, sham + FTE, and I/R model + FTE groups (*n* = 4 each). (**C**) Double immunofluorescence histochemistry of p-ERK (magenta) and IB4 (green) in the T4–T5 dorsal horn following the intracardiac injection of vehicle (left) and 100 μM H_2_O_2_ (right). (**D**) Summary of the number of p-ERK-immunoreactive spinal neurons in lamina I–II per section in the vehicle-treated and H_2_O_2_-treated groups (*n* = 4 each). (**E**,**F**) Double immunofluorescence histochemistry of p-ERK (magenta) and IB4 (green) in the T4–T5 dorsal horn of the I/R, I/R + FTE + vehicle and I/R + FTE + TEMPOL (250 mg/kg) groups, and summary of p-ERK-immunoreactive spinal neurons (*n* = 4 each). (**G**,**H**) Time (**G**) and number of immobility (**H**) during I/R + FTE + vehicle group (*n* = 5) and I/R + FTE + TEMPOL group (*n* = 4); such parameters in rats were recorded in the last 5 min of FTE. All animals were used for the experiments 2 days after the surgery. Data are presented as mean ± SE and examined by unpaired Student’s *t*-test and one-way analysis of variance with Bonferroni analysis. * *p* < 0.05, ** *p* < 0.01. Scale bar = 1 mm (**A**) and 50 μm (**C**,**E**). I/R, ischaemia-reperfusion; ns, not significant; FTE, forced treadmill exercise; DRG, dorsal root ganglion; p-ERK, phosphorylated extracellular signal-regulated kinase; IB4, Isolectin B4; TEMPOL, 4-hydroxy-2,2,6,6-tetramethyl-1-piperidinyloxy; #, number.

H_2_O_2_, a member of ROS, is generated via oxidative phosphorylation in the mitochondria [24]. Considering that H_2_O_2_ contributes to the CAD pathogenesis [25], we examined whether H_2_O_2_ is produced in the heart during FTE. H_2_O_2_ was visualised using a fluorescent indicator (Bes-H_2_O_2_-Ac) via whole-mount cardiac staining. Interestingly, the dense fluorescent signal was distributed in the apex of the left ventricle after FTE in the I/R model group (Figure 3A). Conversely, no fluorescent signals were observed before FTE in this group (Figure 3A). H_2_O_2_ assay showed that FTE significantly increased H_2_O_2_ concentration in the left ventricle area of the I/R model (40.7 ± 1.1 nmol/g, *n* = 4) compared with that of the sham group (33.1 ± 0.5 nmol/g, *n* = 4) or the I/R-without-FTE group (21.7 ± 2.6 nmol/g, *n* = 4) (Figure 3B). To determine whether H_2_O_2_ release induces angina, we confirmed p-ERK expression in the spinal cord after the direct intracardiac injection of 100 μM H_2_O_2_ into the left ventricle. As early as 3 min after the H_2_O_2_ injection, the p-ERK-immunoreactive cells were markedly elevated in the dorsal horn (Figure 3C,D). Therefore, H_2_O_2_ is produced in the heart following physical activity, stimulating nociceptive sensory neurons and mediating angina development.

These findings raised another question, as to whether H_2_O_2_ is involved in angina induced by FTE. To answer this question, we systematically administered the ROS scavenger TEMPOL 30 min before the FTE and evaluated the p-ERK-immunoreactive spinal neurons in the dorsal horn. Although the p-ERK expression was significantly increased by FTE in the vehicle-treated I/R group (7.3 ± 0.9 cells, *n* = 4), TEMPOL significantly suppressed the FTE-induced p-ERK expression (4.3 ± 0.5 cells, *n* = 4) (Figure 3E,F). We did not find any differences in the time (s) and number of immobilities by TEMPOL treatment (Figure 3G,H). Clearly, exercise-induced angina can be provoked by endogenous H_2_O_2_. Thus, this mechanism has a high translational potential to manage angina in patients undergoing post-revascularisation therapy, and ROS may be a critical target for clinical medication.

### 2.4. TRPA1 Channels Expressing Cardiac Sensory Neurons Mediate Cardiac Pain

**Figure 4:**
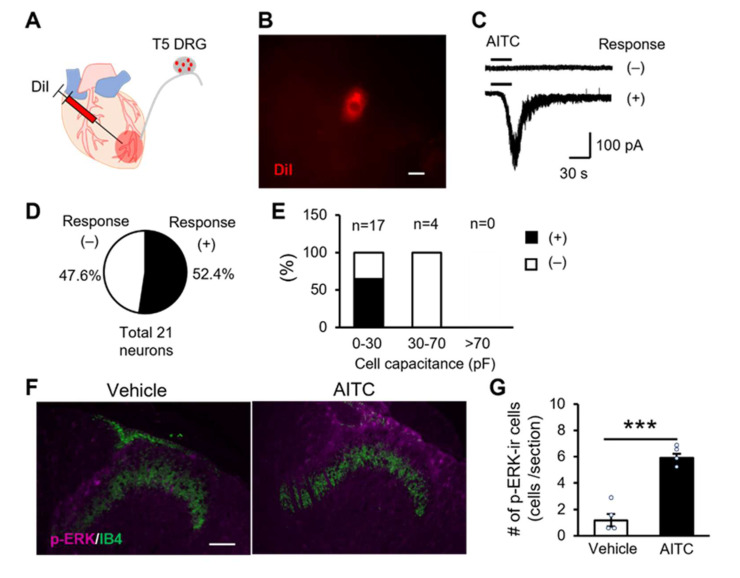
Cardiac sensory neurons express TRPA1 channels that mediate cardiac pain. (**A**) Illustration of the intracardiac injection of DiI in the left ventricle. DIL was injected 7 days before the patch-clamp recording experiments. (**B**) Fluorescent image shows a small-sized DiI-labelled DRG neuron in a whole-mount ex vivo DRG preparation. (**C**) Sample traces of AITC-nonresponsive neurons (top) and AITC-responsive neurons (bottom) following 100 μM AITC bath application under voltage clamp configuration. (**D**) Percentage of AITC sensitivity in DiI-labelled cardiac sensory neurons (*n* = 21). (**E**) Distribution of AITC sensitivity classified by cell capacitance. (**F**) Double immunofluorescence histochemistry of p-ERK (magenta) and IB4 (green) in the T4–T5 dorsal horn following the intracardiac injection of vehicle (left) and 100 μM AITC (right). (**G**) Summary of the number of p-ERK-immunoreactive spinal neurons in lamina I–II per section in the vehicle-treated and AITC-treated groups (*n* = 4 each). Data are presented as mean ± standard error of mean. *** *p* < 0.001, unpaired Student’s *t*-test. Scale bar = 10 μm (**B**) and 50 μm (**F**). DRG, dorsal root ganglion; AITC, allyl isothiocyanate; TRPA1, transient receptor potential ankyrin 1; DiI, 1.1′-dioctadecyl-3,3,3′,3′-tetramethyl-indocarbocyanine perchlorate; p-ERK, phosphorylated extracellular signal-regulated kinase; IB4, Isolectin B4; #, number.

Although we successfully proved the participation of H_2_O_2_ in the process of angina through exercise, the molecular mechanism involved in H_2_O_2_-related cardiac pain in vivo is still unknown. The TRPA1 channel serves as a pain sensor that is activated by various endogenous agonists, including H_2_O_2_ [26]. We hypothesised that the release of H_2_O_2_ from the I/R-injured cardiac tissue stimulates the TRPA1 expression in the cardiac sensory fibres to mediate cardiac pain. To confirm whether cardiac sensory neurons functionally express TRPA1 channels, we conducted a whole-cell patch-clamp on DRG neurons. The cardiac sensory neurons were specifically visualised by injecting DiI, a neural tracer, into the left ventricular wall (Figure 4A). DiI-positive cardiac sensory neurons were identified under fluorescence microscopy, and 21 cardiac sensory neurons were used for the electrophysiological experiment (Figure 4B). AITC, a potent TRPA1-selective agonist, was bath-applied to DiI-positive neurons, and approximately 50% of neurons showed AITC-induced inward currents (Figure 4C,D). Furthermore, small-diameter neurons, which have a whole-cell capacitance of <30 pF, primarily expressed functional TRPA1 channels, but not the medium-diameter neurons (30–70 pF) and large-diameter neurons (>70 pF) (Figure 4E). To determine whether TRPA1 channel activation in the heart evokes cardiac pain in vivo, we directly stimulated the peripheral nerve terminal by injecting AITC into the myocardium. The AITC injection significantly increased p-ERK-immunoreactive spinal neurons in the dorsal horn compared with the vehicle treatment (Figure 4F,G). Therefore, cardiac sensory fibres may respond to endogenous H_2_O_2_ through TRPA1 activation, leading to angina.

**Figure 5:**
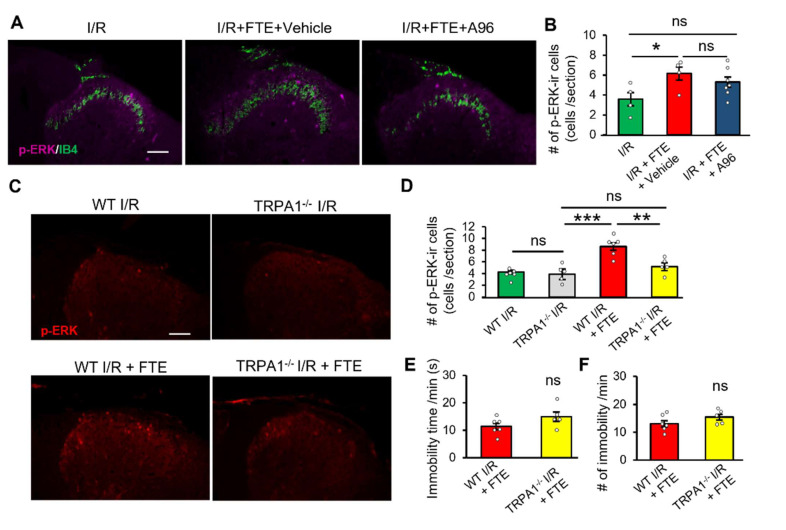
Pharmacological and genetic inhibitions of TRPA1 attenuate exercise-induced angina in I/R model animals. (**A**,**B**) Double immunofluorescence histochemistry of p-ERK (magenta) and IB4 (green) in the T4–T5 dorsal horn of the I/R group (*n* = 4), I/R + FTE + vehicle group (*n* = 4) and I/R + FTE + A96 group (A-967079, 20 mg/kg, *n* = 7), and summary of p-ERK-immunoreactive spinal neurons in rats (*n* = 4 each). (**C**,**D**) Immunofluorescence histochemistry of p-ERK (red) in the T4–T5 dorsal horn of WT I/R group (*n* = 6), TRPA1^−/−^ I/R group (*n* = 5), WT I/R + FTE group (*n* = 6), and TRPA1^−/−^ I/R + FTE group (*n* = 5), and summary of p-ERK-immunoreactive spinal neurons. (**E**,**F**) Time (**E**) and number (**F**) of immobility during FTE in WT I/R + FTE group (*n* = 6) and TRPA1^−/−^ I/R + FTE group (*n* = 5). All animals were used for the experiments 2 days after the surgery. Data are presented as mean ± SE, unpaired Student’s *t*-test and one-way analysis on variance with Bonferroni analysis. * *p* < 0.05, ** *p* < 0.01, *** *p* < 0.001. Scale bar = 50 μm (**A**,**C**). I/R, ischaemia–reperfusion; ns, not significant; FTE, forced treadmill exercise; p-ERK, phosphorylated extracellular signal-regulated kinase; #, number.

Our data demonstrated that H_2_O_2_, an endogenous agonist of TRPA1, causes cardiac pain in naïve animals. To determine whether TRPA1 contributes to angina after FTE, we systemically applied A-967079, a selective TRPA1 antagonist, to the animals 30 min before FTE. Although the vehicle-treated group showed increased p-ERK expression after FTE (6.2 ± 0.6 cells, *n* = 4), systemic administration of A96 inhibited the upregulation of p-ERK after FTE compared with before FTE (5.3 ± 0.5 cells, *n* = 6) (Figure 5A,B). Nevertheless, no difference was found between the vehicle-treated group and the A96-treated group (*p* = 0.34). To further determine the essential role of TRPA1 in angina, we used TRPA1^−/−^ mice for the additional experiment. We built an I/R model on wild-type (WT) and TRPA1^−/−^ mice and applied FTE to evoke angina. Consistent with the results of rats, FTE markedly increased the number of p-ERK-immunoreactive cells in the dorsal horn of the WT I/R model mice. However, the p-ERK expression was suppressed after FTE in the TRPA1^-/-^ I/R group (5.2 ± 0.7 cells, *n* = 5) compared with that in the WT I/R group (8.6 ± 0.6 cells, *n* = 6) (Figure 5C,D). Deletion of TRPA1 channel in TRPA1^-/-^ mice did not affect the time (s) and number of immobility compared with that in the WT mice (Figure 5E,F). Overall, these results strongly suggest that TRPA1 channels, which are expressed on cardiac sensory neurons, are involved in exercise-induced angina development.

## 3. Discussion

Revascularisation therapy is the most effective approach to restore the blood flow of patients with STEMI. Although reperfusion of the coronary artery is important to resuscitate the ischaemic myocardium, it can result in I/R injury. I/R injury may occur following various clinical settings, including thrombolytic therapy and PCI [27,28]. Thus, preventing I/R injury and managing a good quality of life are necessary after such therapies. Approximately 90% of patients with CAD have improved daily movements after receiving PCI; however, 30–40% still experience persistent or recurrent angina [1]. In the present study, the I/R model animals showed a normal daily activity and did not present cardiac ischaemia and pain; however, they presented potential cardiac dysfunctions, increased area at risk, and ECG abnormality. Importantly, these animals had an effort angina, which is a typical clinical symptom of patients with persistent angina following revascularisation therapy. This angina persisted for at least 7 days following I/R, and it was reproducible across different animal species. Therefore, the I/R injury model may be more suitable for studying angina after revascularisation therapy in patients with STEMI.

Angina refers to cardiac pain or discomfort triggered by strenuous activities. Several experimental angina models, such as vasopressin-, methacholine- and isoproterenol-induced angina models, have been used for studying angina [16,17,18]. Further, several indirect indicators such as ST-segment, blood pressure, and biochemical markers of myocardial injury are used to evaluate angina [29,30,31]. Although these hormones or chemicals cause vasospasm, leading to ST-segment deviation, the occurrence of chest pain and ST-segment deviation is not always consistent in human patients [32]. Thus, angina symptoms such as cardiac pain or discomfort have never been studied in animal models. To our knowledge, this study is the first to show a quantified cardiac pain induced by FTE on I/R animal models. ERK, which belongs to the mitogen-activated protein kinase family, is phosphorylated by a noxious peripheral stimulus in the lamina I–II of the dorsal horn [19]. Thus, p-ERK has been used as a marker of neuronal activation following noxious stimulation in pain research to detect somatosensory pain and visceral pain [33]. Therefore, p-ERK could be a useful tool for further study on angina to visualise and quantify cardiac pain in animals.

Generally, ROS are toxic by-products of aerobic metabolism and have been widely implicated in several ischaemic diseases [34]. I/R injury occurs following the restoration of blood flow in the ischaemic tissues, leading to a ‘burst’ of ROS production [35]. The overproduced ROS not only damage tissues by oxidising cellular components but also affect vascular permeability and cell viability [36,37,38]. Although the pathological function of ROS during I/R has been thoroughly investigated, the relationship between ROS and angina is still unclear. In this study, H_2_O_2_ increased in the injured left ventricle following FTE. Furthermore, the scavenging of endogenous H_2_O_2_ after FTE reversed the exercise-induced cardiac pain, and the cardiac injection of exogenous H_2_O_2_ directly evoked cardiac pain. Clearly, H_2_O_2_ has an important role in the development of angina. Although we have not conclusively identified the source of H_2_O_2_ following exercise, one probable source is the myocardium, which generates H_2_O_2_ during low-flow ischaemia through the mitochondrial production or nicotinamide adenine dinucleotide phosphate oxidase-dependent mechanisms [14]. However, we do not have direct evidence whether I/R model animals induce low-flow ischaemia during FTE because of technical limitations of ECG recording from the moving animals. According to a clinical study involving patients with angina, ST-segment depression or vasospasm occurred during exercise, indicating myocardial ischaemia [39]. Considering such evidence, low-flow myocardial ischaemia caused by increased oxygen demand following increased physical activities might trigger H_2_O_2_ production in the I/R-injured myocardium.

To translate our findings into angina management in patients after revascularisation therapy, we suspect that antioxidants may be a promising therapeutic agent for angina that occurs after revascularisation therapy. Edaravone (Radicava), a clinically applicable medication, efficiently scavenges oxygen free radicals by providing a hydrogen atom [40]. In 2017, the U.S. Food and Drug Administration approved edaravone to treat patients with amyotrophic lateral sclerosis (ALS) [41]. Indeed, edaravone has a neuroprotective effect as is thus used for treating acute cerebral ischaemia in Japan [42]. Therefore, antioxidants such as an edaravone may alleviate angina to a great extent by preventing ROS production.

In addition to the generation of angina, H_2_O_2_ is essential for the regulation of coronary blood flow. Thromboxane A2 is a potent vasoconstrictor that mediates coronary vasospasm, and its release from the endothelium is stimulated by H_2_O_2_ [26]. Thus, the inhibition of H_2_O_2_ production may prevent not only angina but also cardiac ischaemia, which is induced by physical activities. Therefore, this study provides a new perspective in the management of angina after revascularisation therapy by preventing H_2_O_2_ or ROS production after physical activities. Additionally, it encourages researchers to further examine whether antioxidants have a potent therapeutic effect on post-PCI patients clinically.

The role of non-neuronal TRPA1 in cardiomyocyte following I/R injury has already been extensively studied. However, the role of TRPA1 on cardiomyocytes remains controversial because TRPA1 activation reportedly has either a cardioprotective or cytotoxic effect in the processing of I/R injury [43,44]. In the present study, we focused on neuronal TRPA1 expressed by cardiac afferents. TRPA1 functions as a pain sensor, thus playing a critical role in pain perception [12]. Approximately 40% of DRG neurons express TRPA1, which is generally limited to small-diameter nociceptive neurons [45]. Consistently, the TRPA1 channel was functionally expressed by approximately 50% of cardiac sensory neurons, and its activation evoked cardiac pain. Therefore, cardiac sensory neurons may detect harmful cardiac abnormalities through TRPA1 activation. H_2_O_2_ is an endogenous agonist of TRPA1 [46], and our study successfully demonstrated its role in angina following exercise. Moreover, pharmacological blocking or gene knockout of TRPA1 significantly attenuated cardiac pain following FTE. A recent study suggested that TRPA1 has no functional expression in both mouse and human cardiomyocytes [47]. Overall, TRPA1 may play a critical role in the generation of exercise-induced angina after a myocardial I/R injury.

H_2_O_2_ has multiple biological activities in physiological and pathophysiological conditions [48,49,50,51]. Thus, H_2_O_2_ stimulates not only the TRPA1 channel but also other H_2_O_2_-sensitive TRP channels, such as TRPV1, TRPM2, and TRPC5 [52,53,54]. Among them, TRPV1 and TRPC5 are involved in pain perception; TRPV1 is activated by noxious heat and acidic pH, whereas TRPC5 is activated by extracellular calcium, nitric oxide, and lipid mediators [55,56]. Therefore, these TRP channels may also be involved in ROS-mediated angina. However, H_2_O_2_-mediated TRPA1 activation has a pivotal role in hypoxia-induced painful dysaesthesia in the hindlimb, and hypoxia-induced pain behaviour was diminished in TRPA1^−/−^ mice rather than in TRPV1^−/−^ mice [57]. Our study revealed that TRPA1^−/−^ mice displayed a completed anti-angina effect following exercise. Meanwhile, TRPC5 is involved in inflammatory pain in various disease models [58]; hence, this channel may contribute to cardiac inflammatory pain such as in endocarditis, myocarditis, and pericarditis, instead of contributing to ischaemic pain. Taken together, the TRPA1 channel mediates angina following exercise, suggesting a possible molecular mechanism of angina after revascularisation therapy.

## 4. Materials and Methods

### 4.1. Animals

Male Sprague Dawley rats at 7 weeks old and C57BL/6 mice at 10 weeks old were purchased from SLC Inc. (Shizuoka, Japan). These rats and mice were housed in plastic cages on a reversed light cycle (12 h light/12 h dark cycle) with water/food provided ad libitum in a temperature-controlled animal facility (23 °C ± 1 °C). Makoto Tominaga (Okazaki Institute for Integrative Bioscience, Okazaki, Japan) provided TRPA1-deficient mice, which were originally produced by David Julius (University of California, San Francisco, CA, USA). The Hyogo University of Health Sciences Committee on Animal Research approved all our animal experimental procedures, which conformed to the NIH Guide for the Care and Use of Laboratory Animals.

### 4.2. Myocardial I/R Injury Model

Preoperatively, the rats or mice were anaesthetised by injecting medetomidine, midazolam, and butorphanol (0.15 or 0.3 mg/kg, 2 or 4 mg/kg, and 2.5 or 5 mg/kg, respectively) intraperitoneally. They were intubated and mechanically ventilated using a rodent respirator device (NARCOBIT KN-472, Natsume Seisakusho Co., Tokyo, Japan); the ventilation rate and volume were 85–90 bpm and 500 mL/min for the rats and 95–100 bpm and 300 mL/min for the mice. The animals underwent thoracotomy at the left fourth intercostal space to expose the heart. Ischaemia was achieved by left anterior descending coronary artery (LAD) ligation with PE-10 tubing (Becton Dickinson, Tokyo, Japan) using a silk suture (6-0 or 7-0; Natsume Seisakusho Co., Tokyo, Japan). Reperfusion was achieved by releasing the ligature 30 min after occlusion. The same procedure was applied for the sham surgery, except for the LAD ligation. The air in the thoracic cavity was removed using a syringe, and the intercostal space, muscles, and skin were sutured with 3-0 silk. The model rats and mice were used for experiments 2 or 7 days after reperfusion.

### 4.3. Intracardiac Injection

Rats were anaesthetised and ventilated in the same processes described above. To avoid the effect of surgical invasion, we incised the skin from the right side of the chest for thoracic cavity exposure. Using a Hamilton microsyringe (72–1001, Sansyo Co., Tokyo, Japan), we injected 20 μL of allyl isothiocyanate (AITC, 100 μM) (5% dimethyl sulfoxide [DMSO] in saline) or 100 μM of hydrogen peroxide (in saline) into the cardiac muscle of the left ventricle. After 3 min from the intracardiac injection, we fixed the animals by 4% paraformaldehyde (PFA) for immunohistochemical analysis. For the retrograde labelling of cardiac sensory neurons, 5 μL of 4% Fluoro-Ruby (in saline, Fluorochrome, Denver, CO, USA) or 4% of 1.1′-dioctadecyl-3,3,3′,3′-tetramethyl-indocarbocyanine perchlorate (DiI) (in 5% DMSO in saline) was injected into the cardiac muscle of the left ventricle. The rats were used for immunohistochemical analysis or patch-clamp experiment 7 days after the injection.

### 4.4. Electrocardiography

The rats inhaled 2.5% isoflurane (Fujifilm Co., Osaka, Japan) and were placed in a supine position. The cardiogram waveform was monitored by a 3-point guidance method. We placed the negative electrode, positive electrode, and earth electrode on the right front paw, left hind paw, and left front paw, respectively. The electrical signal was recorded at 1 kHz using an amplifier (Bio Amp FE232; ADInstruments, Dunedin, New Zealand). Furthermore, the ECG waveform was analysed using a specific software (ECG Analysis Module V8, LabChart Pro, ADInstruments, Dunedin, New Zealand).

### 4.5. FTE

After 2 days postoperatively, FTE experiments were conducted using the I/R model or sham rats with an animal treadmill apparatus (TMS-2B, MELQUEST Co., Toyama, Japan). We set the exercise intensity according to the animal’s maximal oxygen uptake and applied a speed of 20 m/min for rats or 15 m/min for mice for 10 min. During the FTE, the animals’ behaviour was monitored using a camera (HDR-CX470, SONY, Tokyo, Japan). The time (s) and number of immobility (rest) were analysed during the last 5 min of FTE. After FTE, we immediately fixed with 4% PFA within 3 min.

### 4.6. Drug Application

Drugs were systemically administered via intravenous infusion through a catheter 30 min before the FTE. We used 1 mL of 4-hydroxy-2,2,6,6-tetramethyl-1-piperidinyloxy (TEMPOL) (250 mg/kg, H0865; Tokyo Chemical Industry Co., Ltd., Tokyo, Japan) and its vehicle (0.9% saline), or A967079 (A96, 20 mg/kg, A-225, Alomone Labs) and its vehicle (10% DMSO, 5% Tween 80, 0.5% methylcellulose).

### 4.7. Perfusion Fixation through the Abdominal Aorta

To avoid chest pain from the surgical manipulation for PFA perfusion, we conducted the PFA perfusion via the abdominal aorta. A 22 G (for rats) or 30 G (for mice) needle was inserted into the abdominal aorta and perfused with 1% PFA in 0.1 M phosphate buffer (PB), followed by 4% PFA in 0.1 M PB. In some experiments, the rats were fixed transcardially to dissect out thoracic spinal cord and thoracic DRGs. The samples were post-fixed with 4% PFA in 0.1 M PB at 4 °C overnight and embedded using the O.C.T compound (4583; Sakura Finetek Japan Co., Tokyo, Japan) and frozen by powdered dry ice.

### 4.8. Immunohistochemistry

The samples were sectioned by cryostat (NX70; Thermo Fisher Scientific Inc., MA, USA) at 25 μm of free-floating sections for rats or 14 μm of mounting sections for mice. The sections were incubated with a blocking buffer (0.1 M TBS with 0.5% Tween-20) in 10% normal donkey or goat serum with 0.1 M tris-buffered saline (TBS, pH = 7.4) and 0.5% Tween-20 for 1 h at room temperature. The sections were applied with anti-p-ERK (1:500, Cat# 4370s, RRID: AB_2315112) and Isolectin B4 (IB4, 1:500, Cat# L2140, RRID: AB_2313663) in 5% serum in 0.1 M TBS with 0.5% Tween-20 at 4 °C overnight. We incubated the sections with Alexa Fluor 594-conjugtated goat anti-donkey (1:1000, Cat# A-21207, RRID: AB_141637) or Alexa Fluor 594-conjugtated donkey anti-ribbit antibody (1:1000, Cat# A11037, RRID: AB_2576217) and Alexa Fluor 488-conjugated streptavidin (1:1000, Cat# S-11223, RRID: AB_2336881) for 1 h at room temperature. The sections were mounted with an anti-fade reagent (H-1200; Vector Labs, Burlingame, CA, USA). Images were obtained using a microscope (Eclipse 80i; Nikon Instruments Inc., Tokyo, Japan) equipped with a digital camera and operated by NIS-Elements D 3.2 software (RRID: SCR_014329). IB4 immunoreactivity was used as an indicator of inner lamina II of the superficial dorsal horn. In quantifying the number of p-ERK-immunoreactive cells in the lamina I–II layers, four or five sections per animal were used for the analysis using ImageJ software (RRID: SCR_003070).

### 4.9. Patch-Clamp Recording

One week after retrograde labelling with DiI, T4–T5 DRGs were dissected out and affixed in a recording chamber by a tissue anchor and submerged in a Krebs solution that contained the following (in mM): 117 NaCl, 3.5 KCl, 2.5 CaCl_2_, 1.2 MgCl_2_, 1.2 NaH_2_PO_4_, 25 NaHCO_3,_ and 11 glucose. In addition, pH was adjusted to 7.35 with NaOH, and osmolarity was adjusted to 324 mOsm with sucrose. The DRGs were treated by a mixture of 0.07% dispase II (Godo Shusei Co., Tokyo, Japan) and 0.07% collagenase (Nacalai Tesque, Kyoto, Japan) in Krebs solution for 5 min at room temperature.

DiI-positive cardiac sensory neurons were visualised under a 40× (NA 0.80) objective and with CCD camera (C4742-80; Hamamatsu Photonics K.K., Shizuoka, Japan). Recording electrodes were filled with an internal solution containing the following (in mM): 105 K-gluconate, 30 KCl, 0.5 CaCl_2_, 2.4 MgCl_2_, 5 EGTA, 10 HEPES, 5 Na_2_ATP, and 0.33 GTP-TRIS salt; the pH was adjusted to 7.35 with KOH, and osmolality was adjusted to 330 mOsm with sucrose; the electrode resistance was 5 MΩ. After the whole-cell mode was established, the cell was held at −60 mV. The signals were amplified using an Axopach 200B amplifier, filtered at 2 kHz, and sampled at 10 kHz using the pCLAMP 10 software (MOLECULAR DEVICES, San Jose, CA, USA).

### 4.10. Hydrogen Peroxide Assay

After the rats were anaesthetised with isoflurane, we collected 0.2 g of heart from four groups (sham, I/R model, sham with 10 min FTE and I/R with 10 min FTE). The samples were homogenised in 0.7 mL of saline, and the supernatant was corrected after the centrifuge (15,000× *g* at 4 °C for 15 min). The concentration of hydrogen peroxide assay was analysed with Bioxytech H_2_O_2_ 560^TM^ Quantitative Peroxidase Assay Kit (CL-204; Cosmo Bio Co., Tokyo, Japan) In addition, the concentration of each sample was read by a spectrophotometer (Molecular Devices, San Jose, CA, USA) at 560 nm.

### 4.11. Assessment of Myocardial Infarct Size and the Risk Area

The myocardial infarct size and risk area of the heart were measured by combining Evans blue (EB, 054-0406; Fujifilm Wako Chemicals Co., Osaka, Japan) and 2,3,5-triphenyltetrazolium chloride (TTC, 205-05833; Fujifilm Wako Chemicals Co., Osaka, Japan). Two days after the surgery, 1% EB (in 0.9% saline, 5 mL/kg) was injected via the lateral tail vein. Within 2–3 min, they were sacrificed, and their hearts were removed, which were then placed in the freezer at −30 °C for 1 h. The frozen hearts were incised into five 1–2 mm-thick parallel transverse slices, incubated in 1% TTC (in 0.9% saline) at 37 °C for 15 min, and post-fixed with 4% formalin solution for 10 min. The infarct size and risk area were evaluated by ImageJ (RRID: SCR_003070).

### 4.12. Recording of Daily Animal Activity

Two days after the I/R surgery, the rats were placed in plastic cages individually on a reversed light cycle (12 h light/ 12 h dark cycle) with water/food provided ad libitum in a temperature-controlled animal facility (23 °C ± 1 °C). The cages were placed under the infrared detectors connected to a telemetry transmitter (DAS64; Neuroscience Inc., Tokyo, Japan). Animal activities were counted for 24 h using an activity measurement tool (Act-1 software; Nikon Co, Tokyo, Japan).

### 4.13. Statistics

All statistical data were analysed using Prism software (version 7; GRAPH PAD, San Diego, CA, USA). For each group, differences were assessed using one-way analysis of variance with Bonferroni analysis. Two groups were compared by Student’s *t*-test. All the statistical results are represented as mean ± standard error of mean. Differences were considered to be significant if * *p* < 0.05, ** *p* < 0.01, and *** *p* < 0.001.

## 5. Conclusions

To the best of our knowledge, this study provided the first evidence of exercise-induced angina following myocardial I/R in rats. We found that the production of H_2_O_2_ through physical exercise contributes to the angina development following I/R. ROS may provide a desirable pharmacological target for the clinical treatment of post-PCI angina. Considering that the TRPA1-selective antagonist is not a clinically applicable medication, free radical scavengers might have the potential to treat patients with angina after revascularisation therapy.

## Figures and Tables

**Table 1:** ECG parameters of sham and I/R groups.

	Sham	I/R	*p*-Value ^a^
Sample size, *n*	6	8	
Heart Rate (BPM)	421.0 ± 8.4	407.0 ±7.0	0.25
RR Interval (ms)	142.8 ± 3.0	147.8 ± 2.7	0.28
P Duration (ms)	20.0 ± 1.0	24.0 ± 1.5	0.08
QRS Interval (ms)	13.0 ± 1.0	11 ± 0.9	0.22
QT Interval (ms)	63.4 ± 3.0	40.6 ± 8.5	0.06
Q Amplitude (µV)	12.0 ± 2.0	51.1 ± 23.2	0.20

All animals were used for the experiment 2 days after surgery. Abbreviation: BPM, beats per minute. ^a^: Data are presented as mean ± standard error of mean. Unpaired Student’s *t*-test.

## Data Availability

The data underlying this article will be shared on reasonable request to the corresponding author.

## Supplementary Materials

The following supporting information can be downloaded at: https://www.mdpi.com/article/10.3390/ijms23052820/s1.

## Abbreviations

PCI	Percutaneous coronary intervention
CAD	Coronary artery disease
STEMI	ST-segment elevation myocardial infarction
DRG	Dorsal root ganglion
TRPA1	Transient receptor potential ankyrin-1
p-ERK	Phosphorylated extracellular signal-regulated kinase
FTE	Forced treadmill exercise
ECG	Electrocardiogram
