# Supplementary material for: Reactive Oxygen Species Cause Exercise-Induced Angina in a Myocardial Ischaemia-Reperfusion Injury Model
Source: Int J Mol Sci. 2022 Mar 4;23(5):2820. doi: 10.3390/ijms23052820 (PMC8910887; doi:10.3390/ijms23052820)
Supplement: Supplementary file 1 [file ijms-23-02820-s001.zip › ijms-1610629-supplementary.pdf]

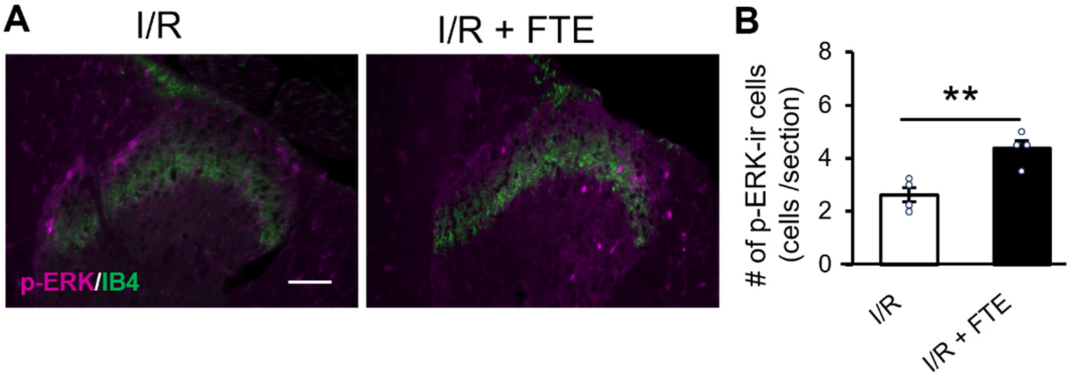

Figure S1. Forced treadmill exercise evokes angina in I/R model rats 7 days after surgery. **(A,B)** Double immunofluorescence histochemistry of p-ERK (magenta) and IB4 (green) in the T4–T5 dorsal horn of the I/R group and I/R + FTE group, and summary of p-ERK-immunoreactive spinal neurons in rats ($n = 4$ each). All animals were used for the experiments 7 days after the surgery. Data are presented as mean ± SE, unpaired Student's *t*-test. ** $p < 0.01$. Scale bar = 50 μm (A). I/R, ischaemia–reperfusion; FTE, forced treadmill exercise; p-ERK, phosphorylated extracellular signal-regulated kinase; #, number.
